# Insight into the molecular mechanism of miR-192 regulating *Escherichia coli* resistance in piglets

**DOI:** 10.1042/BSR20171160

**Published:** 2018-02-21

**Authors:** Li Sun, Sen Wu, Chao-Hui Dai, Shou-Yong Sun, Guo-Qiang Zhu, Sheng-Long Wu, Wen-Bin Bao

**Affiliations:** 1 Key Laboratory for Animal Genetics, Breeding, Reproduction and Molecular Design, College of Animal Science and Technology, Yangzhou University, Yangzhou 225009, China; 2 College of Veterinary Medicine, Yangzhou University, Yangzhou 225009, China; 3 Joint International Research Laboratory of Agriculture and Agri-Product Safety, Yangzhou University, Yangzhou 225009, China

**Keywords:** Escherichia coli, intestinal epithelial cell line, miRNA, swine, TALEN

## Abstract

MicroRNAs (miRNAs) have important roles in many cellular processes, including cell proliferation, growth and development, and disease control. Previous study demonstrated that the expression of two highly homologous miRNAs (miR-192 and miR-215) was up-regulated in weaned piglets with *Escherichia coli* F18 infection. However, the potential molecular mechanism of miR-192 in regulating *E. coli* infection remains unclear in pigs. In the present study, we analyzed the relationship between level of miR-192 and degree of *E. coli* resistance using transcription activator-like effector nuclease (TALEN), *in vitro* bacterial adhesion assays, and target genes research. A TALEN expression vector that specifically recognizes the pig miR-192 was constructed and then monoclonal epithelial cells defective in miR-192 were established. We found that miR-192 knockout led to enhance the adhesion ability of the *E. coli* strains F18ab, F18ac and K88ac, meanwhile increase the expression of target genes (*DLG5* and *ALCAM*) by qPCR and Western blotting analysis. The results suggested that miR-192 and its key target genes (*DLG5* and *ALCAM*) could have a key role in *E. coli* infection. Based on our findings, we propose that further investigation of miR-192 function is likely to lead to insights into the molecular mechanisms of *E. coli* infection.

## Introduction

MicroRNAs (miRNAs) are a class of short noncoding RNA molecules of approximately 22 nucleotides in length that are widely found in animals and plants. MiRNAs are able to post-transcriptionally regulate gene expression, either by the degradation of the targeting mRNAs or by the inhibition of normal translation through the complete or incomplete pairing with the 3′-untranslated region (3′-UTR) [1]. Most miRNA studies have focused on human and some model animals (such as zebrafish and mice), with few miRNA studies having been conducted in swine. Although the miRbase database currently contains 326 porcine mature miRNA sequences, the majority of these miRNA have yet to be assigned a function. Recent advances in high-throughput sequencing technologies have made direct sequencing of porcine miRNA possible, leading to the discovery of many novel miRNAs. MiRNAs have recently been implicated in regulating the course of porcine reproductive and respiratory syndrome, as well as viral and bacterial diseases, such as H1N1 influenza and salmonellosis [2–4]. Using high-throughput sequencing technology, we previously identified two miRNAs (miR-192 and miR-215) differentially expressed between *Escherichia coli* F18 resistant and sensitive duodenum (NCBI database Gene Expression Omnibus, GEO Series accession number GSE32527) [5]. MiR-192 and miR-215 are highly homologous miRNAs that have been proposed as positive regulator proteins of the tumor suppressor gene p53 [6]. As such, miR-192 and miR-215 are potential biomarkers and drug targets [7]. However, the regulatory mechanism of miR-192 and miR-215 in porcine disease is as yet unclear.

Sharbati et al. [8] found high expression levels of miR-215 and miR-192 in rat duodenum and jejunum, while Mckenna et al. [9] found that miR-192 expression was the most abundant among 453 miRNA families identified in the intestinal mucosa of Dicer1 mutated mice. Wu et al. [10] also found that miR-192 and miR-215 are highly expressed in the duodenum and jejunum of 35-day-old Meishan piglets. In conclusion, the above evidence suggest a probable important regulatory function for miR-192 and miR-215 in the porcine intestine.

Enterotoxigenic *E. coli* (ETEC) are the main pathogens responsible for diarrhea in newborn and weaned piglets. ETEC binds to receptors of the piglet small intestinal epithelial cells brush border through adherence to pili cells, leading to the production of enterotoxin and ultimately to diarrhea. Thus, ETEC pathogenicity is dependent on the expression of the corresponding receptors in the piglet small intestinal epithelial cell brush border and the adherence of ETEC to these receptors [11].

In the present study, we investigated the functions of miR-192 using a gene knockout approach and target genes research. We predicted five target genes (*ALCAM, DLG5, MIPOL1, FRMD4B*, and *ZFHX3*) of miR-192 related to *E. coli* and studied the influence miR-192 in target genes. MiR-192 knockout was achieved using the recently developed transcriptional activator-like effector nuclease (TALEN) targeted gene editing technology, which allows specific recognition and cleavage of the target DNA region [12–14]. The present study will explore the effects of miR-192 knockout on the expression of the target genes and the adhesion of *E. coli*. These results will provide insights into the concretely regulatory mechanisms of miR-192 in porcine intestinal epithelial cell adhesion and *E. coli* resistance, and provide a basis for screening and obtaining effective markers for disease-resistance breeding.

## Materials and methods

### Ethical statement

The animal study proposal was approved by the Institutional Animal Care and Use Committee (IACUC) of the Yangzhou University Animal Experiments Ethics Committee (permit number: SYXK(Su) 2012-0029). All experimental procedures were performed in accordance with the Regulations for the Administration of Affairs Concerning Experimental Animals approved by the State Council of the People’s Republic of China.

### Experimental reagents

The TALEN Assembly Kit, including the L14, L16, and L17 left arm TALEN backbone vectors and R10 right arm TALEN backbone vector, were purchased from Sidansai Biotechnology (Shanghai, China). Kanamycin and puromycin were purchased from Sigma-Aldrich (U.S.A.); DMEM (Dulbecco’s modified eagle medium), F12 (Ham’s F12 nutrient medium), Opti-MEM cell culture medium, and fetal bovine serum were purchased from Gibco (U.S.A.). *Trans*IT-LT1 transfection reagents were purchased from Mirus Corporation (U.S.A.). The Reverse Transcription Kit, SYBR Quantification Kit and Plasmid Extraction Kit were purchased from Takara Company (Dalian, China). Restriction endonuclease *BamH*I and *Pst*I were purchased from Fermentas (Canada). The porcine intestinal epithelial cell line IPEC-J2 was a kind gift from Dieter Schifferli (University of Pennsylvania). The *E. coli* F18ab, F18ac, and K88ac were cultivated in our laboratory.

### Experimental animals

Previously, our research group established the *E. coli* F18 disease-resistant and the *E. coli* F18-susceptible resource populations from Suzhou Sutai Pig Breeding Centre, China. In the present study, eight *E. coli* F18-resistant piglets and eight *E. coli* F18-susceptible piglets were strictly identified and obtained via the aforementioned verified *E. coli* F18-resistant and susceptible individuals. Piglets which were raised in the same environment were killed at postweaning days (35 days). Duodenum tissues were taken and stored in liquid nitrogen on-site, and then transferred to a −70°C freezer.

### TALEN recognition sequence design

**Figure 1:**
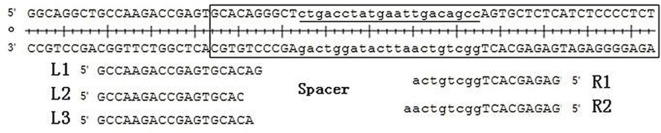
Scheme of the TALEN recognition sequence The figure shows the three left arm targets (L1, L2, and L3), the two targets right arm targets (R1 and R2), miR-192 precursor sequence (boxed) and miR-192 mature sequence (underlined), and there was a spacer approximately 20 bp between left and right arms.

The porcine miR-192 precursor is approximately 80 bp long (accession: NR_038549.1). The recognition sequence targeting the right and left arms of the porcine TALEN mature body was designed according to the pig genome sequence using online design software (https://tale-nt.cac.cornell.edu/). The three left arm targets (L1, L2, and L3) and the two right arm targets (R1 and R2) resulted in a total of six TALEN combinations (L1R1, L1R2, L2R1, L2R2, L3R1, and L3R2). The appropriately matched groups were selected according to the recognition sequence composition and the desired vector was chosen based on the final base of the recognition sequence (Table 1 and Figure 1). The primers containing TALEN knockout locus (miR-192 maturation sequence region) were designed as: forward primer, 5′-CCTGTAACAGCAACTCCAT-3′; reverse primer, 5′-GGCATTGAGGCGAACATA-3′. The targeted amplification fragment was predicted to be 287 bp. All primers were synthesized by Shanghai Biological Engineering Technology Co. Ltd.

**Table 1:** Recognition sequences of the TALEN left and right arms

Name		Recognition sequence	Assembling	Vector
Left arm	L1	GCCAAGACCGAGTGCACAG	GC1CA2AG3AC4CG5AG6TG7CA8CA9	L17
	L2	GCCAAGACCGAGTGCAC	GC1CA2A3GA4CC5G6AG7TG8CA9	L16
	L3	GCCAAGACCGAGTGCACA	G1CC2AA3GA4CC5GA6GT7GC8AC9	L14
Right arm	R1	GAGAGCACTGGCTGTCA	GA1G2AG3CA4CT5G6GC7TG8TC9	R10
	R2	GAGAGCACTGGCTGTCAA	GA1GA2GC3A4CT5GG6CT7GT8CA9	R10

### TALEN left and right arm vector assembly, connection, and identification

Each corresponding numbered module (1.5 μl) was transferred from the kit to a corresponding microcentrifuge tube based on the design recognition sequence and matching scheme (Table 1), followed by 1.5 μl of the corresponding TALEN backbone vector, 2.0 μl of solution 3, 1.0 μl of solution 1, 1.0 μl of solution 2, and ddH_2_O to a final volume of 20 μl. In which, solutions 1, 2, and 3 were used for ligation reaction of TALEN vectors of left and right arms. The reaction was performed in a thermocycler as follows: 37°C for 5 min, 16°C for 10 min, 15 cycles; 80°C for 10 min and 12°C for 1 min. Next, 1.0 μl of solution 4 and 0.5 μl of solution 5 were added to the microcentrifuge tubes, followed by incubation at 37°C for 1 h. In which, solutions 4 and 5 were used for strengthening the following transformation assays. The final product was transformed into competent *E. coli*, which was plated onto Luria-Bertani (LB) agarose plate containing kanamycin (100 μg/ml). After incubation at 37°C overnight, ten monoclones were picked and inoculated into LB broth containing kanamycin and cultured at 37°C in a shaking incubator (250 r/min) for 12 h, followed by plasmid extraction.

Plasmid (2.0 μl), Buffer G (1.0 μl), *BamH*I (1 U), *Pst*I (1 U), and ddH_2_O (to a final volume of 10 μl) were added to the double digestion system and incubated at 37°C for 3 h. Digested plasmids of the target length were sequenced using the sense primer 5′-CTCCCCTTCAGCTGGACAC-3′ and antisense primer 5′-AGCTGGGCCACGATTGAC-3′. Proper construction of the TALEN vector was confirmed by sequence alignment against the standard sequence using tblastx (http://blast.ncbi.nlm.nih.gov/Blast.cgi).

### Minimum lethal concentration for puromycin

IPEC-J2 cells were cultured with complete medium (1:1 ratio of DMEM and F12, plus 10% fetal bovine serum) and supplemented with puromycin at concentrations of 1, 2, 3, 4, and 5 μg/ml during the exponential growth phase. The medium was replaced with fresh medium containing the corresponding concentration of puromycin every 24 h and the puromycin concentration at which all cells died after 3 days was defined as the minimum lethal concentration, which could then be used for resistance screening after transfection.

### Cotransfection of IPEC-J2 cells with TALEN left and right arm plasmid vectors

IPEC-J2 cells were cotransfected with the extracted TALEN left and right arm plasmid vectors and cultured in six-well plates. The culture medium was replaced with 2.5 ml of fresh complete medium when the IPEC-J2 cells reached 70% confluence. Plasmid mixtures (2.5 μg, six combinations, with an equal ratio of the left arm and right arm vectors) were added to 250 μl of Opti-MEM and gently mixed. TransIT-LT1 transfection reagent (7.5 μl) was then added and gently mixed. The solution was then added to cell culture plates and, after 20 min at room temperature, was mixed by gentle shaking and transferred to a culture incubator. A control group with no plasmid was set up in parallel.

### Puromycin screening and identification

At 24 and 48 h after transfection, the media were refreshed with complete medium containing the puromycin minimum lethal concentration. At 72 h after transfection, the media were replaced with complete medium without puromycin. The cells were then digested and collected. To confirm the TALEN knockout efficiency, aliquots of these samples were used for DNA extraction and PCR sequencing. The amplified PCR product containing the targeted fragment length was sequenced and the overlap of the sequence peak was observed. The sequencing results were then aligned with the pig genome sequence to confirm that the amplified fragment was correct and that TALEN mediated knockout had been achieved. The remaining cells were used to establish monoclonal cell culture lines.

### Monoclonal cells selection and identification

After puromycin screening, cells with correct targeting were selected and used to establish monoclonal cells. The cell suspensions were diluted to a concentration of less than 1 cell per 100 μl and 100 μl of these diluted cell suspensions were seeded into each well of 96-well plates. After the cells had successfully adhered to the culture plates within a CO_2_ incubator, wells containing monoclonal cells were monitored and labeled; wells judged to contain more than a single original cell were removed. Following growth of the monoclonal cells into cell populations, cells were digested, passaged to 12-well plates, and collected after culturing to full confluence. These cells were used for passage and DNA extraction. The extracted DNA was subsequently used as template for PCR and sequencing. Samples with an overlapped peak in the sequencing process were connected with a T vector, and also executed with monoclonal sequencing. Secondary conformational analysis was used for the miR-192 precursor sequence with base deficiency using the RNA structure software (version 5.6) [15]. The integral workflow of producing the intestinal epithelial cells with miR-192 knockout through TALEN technology was as described in Supplementary Figure S1.

### Target genes prediction and screening of miR-192 related to *E. coli* F18 resistance

Because there is no suitable database and method for target genes prediction of porcine miRNA, so porcine miR-192 is aligned to human miRNA according to sequence, and then we predicted its target genes through the human TargetScan database (http://www.targetscan.org/). Based on the intersection between predicted target genes and the differential expressed genes identified in our previous study [16], we further screened out some key target genes related to weaned piglets *E. coli* F18 resistance.

### MiR-192 host gene and partial target gene transcription detection

Cross-intron quantitative real-time PCR (qRT-PCR) primers of host gene and partial target genes were designed to investigate the effects of miR-192 knockout on the formation and expression of the gene transcript, and inquire into the different expression of target genes between the *E. coli* F18-resistant and susceptible population in Sutai pigs (Table 2). Reverse transcribed cDNA (positive monoclonal group, control group cells, and duodenum tissues) was used as template. The product was identified by consistency with the targeting fragment according to 12% polyacrylamide gel electrophoresis, while the base composition was detected by direct sequencing reactions. The expression level of the transcript was assessed by qRT-PCR as follows: 10 μl of SYBR Premix ExTap^TM^ II (2×), 0.4 μl for each primer (10 μM), 0.4 μl of ROX Reference Dye II (50×), and 2 μl of DNA template in a 20 μl of reaction volume, with a thermocycler program of 40 cycles of 95°C for 30 s, 95°C for 5 s, and 60°C for 34 s. To analyze the specificity of the amplified products, melting curve analysis was executed after amplification as follows: 95°C for 15 s, 60°C for 1 min; 95°C for 15 s, and 60°C for 15 s.

**Table 2:** Primer sequences of quantitative real-time PCR

Name	Sequence (5′-3′)	Length of sequence/bp	Accession no.
*P*-F	GACCCTACTTCCTCCTTGTGCC	110	LOC100519466
*P*-R	GGACTTTGCCCTCAAGGAGTCT		
*ALCAM*-F	CTGGCAGTGGAAGCGTCATA	123	XM_003358827.3
*ALCAM*-R	GCTGGTTTTCTGCTGTGCAA		
*DLG5*-F	ATCCCTCTGTCATCGACCCA	185	XM_005671132.1
*DLG5*-R	GTGCAGGTTCCCACCACATA		
*FRMD4B*-F	TGGAAACTGGAGGACCCAGA	189	XM_003132316.2
*FRMD4B*-R	TGCTCACTACTCTCCAGGCT		
*MIPOL1*-F	CAATGCAGACACAGGGATAGC	154	XM_005666198.1
*MIPOL1*-R	TAGCCGTTTGCACTGAGACA		
*ZFHX3*-F	AGATCGAGACGGGAACTCCA	250	XM_005655766.1
*ZFHX3*-R	CTGAGGCCTCTTGGCATGAA		
*GAPDH-F*	ACATCATCCCTGCTTCTACTGG	188	NM_001206359.1
*GAPDH-R*	CTCGGACGCCTGCTTCAC		

### Western blotting verification

Total proteins were extracted using an NE-PER kit (Nuclear and Cytoplasmic Extraction Reagents, Thermo Fisher Scientific Inc.) according to the manufacturer’s protocol. Protein levels were normalized using a BCA kit (Thermo Fisher Scientific Inc.). SDS/PAGE conditions were 10 μl of protein loaded to a 10% gel run at 120 V for 90 min. For Western blotting, proteins were transferred to PVDF membranes and immunoblotted with primary antibodies against DLG5 (1:600), ALCAM (1:800), and β-actin (1:4000) (Abcam, Cambridge, MA, U.S.A.). The secondary antibody was horseradish peroxidase (HRP) conjugated goat anti-rabbit IgG (Jackson ImmunoResearch Laboratories, West Grove, PA, U.S.A., 1:5000).

### *E. coli* adhesion level verification

Cells were seeded in 12-well plates at a density of 5 × 10^5^ and cultured until full confluence. *E. coli* F18ab, F18ac, and K88ac were inoculated in LB medium and cultured for 12 h with 200 r/min shaking. The bacteria were collected by centrifugation at 3000 r/min for 10 min and washed with PBS (phosphate buffer saline) buffer three times. Bacteria were diluted to 1 × 10^9^ CFU/ml with cell culture medium and 1 ml of the bacteria suspension added to well-plates in triplicate, followed by incubation for 1 h at 37°C in 5% CO_2_. Medium containing bacteria was discarded and each well was washed three times with PBS. DNA extraction kit lysate was then immediately added and DNA extracted according to the manufacturer’s instructions.

The extracted mixed bacterial DNA was then used as template for qRT-PCR in triplicate for each sample. The primers used for detecting adhesion level were shown in Table 3 [17]. The results were analyzed by the 2^−ΔΔ*C*^_t_ method [18] using β-ACTIN as the reference gene as follows: ΔΔ*C*_t_ = bacterial gene *C*_t_ values − cell gene *C*_t_ values; ΔΔ*C*_t_ = Δ*C*_t_ values of knockout group − Δ*C*_t_ values of the control group.

**Table 3:** Primer sequences of the fimbriae and β*-ACTIN* genes used in real-time PCR

Name	Sequence (5′-3′)	Length (bp)	Accession no.
*PILIN*-F	AGGCCGAACCAAAGAAGCAT	117	M25302.1
*PILIN*-R	TCACCATCAGGGTTTCTGAGT		
β*-ACTIN*-F	GTCGTACTCCTGCTTGCTGAT	119	NC_010445.3
β*-ACTIN*-R	CCTTCTCCTTCCAGATCATCGC		

### Data statistics and analysis

The relative quantitative results were processed using 2^−ΔΔ*C*^_t_ method. The analysis used the following formula: ΔΔ*C*_t_ = (average *C*_t_ value of target gene from tested group − average *C*_t_ value of reference gene from tested group) − (average *C*_t_ value of target gene from control group − average *C*_t_ value of reference gene from control group). The general linear model in SPSS 20 software was used to compare the expression of miR215, the expression of target genes, and bacteria adhesion of cells before and after transfection.

## Results

### Validation of TALEN vector

**Figure 2:**
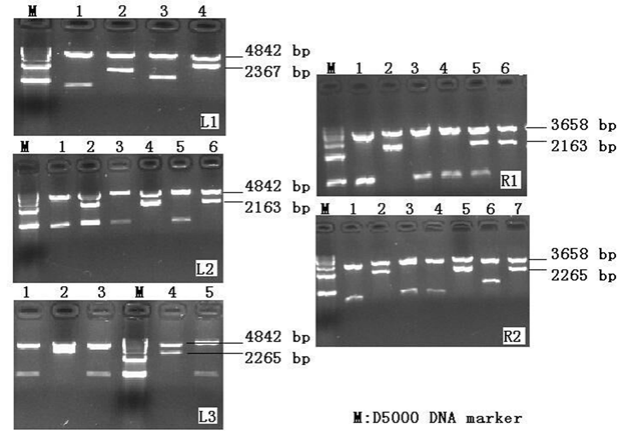
TALEN left (L1, L2, and L3) and right arm target (R1 and R2) assembly vectors by double-enzyme digestion electrophoresis M represents D5000 DNA Marker (Takara Biomedical Technology (Beijing) Co., Ltd.). The L14, L16, and L17 left arm TALEN backbone vectors were 5373 bp, and R10 right arm TALEN backbone vector was 4189 bp. “ 4” at Figure L1; “ 4”, “ 6” at Figure L2; “ 2”, “ 4” at Figure L3; “ 2”, “ 6” at Figure R1; and “ 2”, “ 5”, “ 6” at Figure R2 were TALEN vectors which were successfully constructed. Others were unsuccessfully constructed. All vectors which were consistent with the analysis process were positive clone vectors.

To ensure that the vectors were successfully constructed, we performed restriction enzyme digestion of the vectors with *BamH*I and *Pst*I. The L14, L16, and L17 left arm TALEN backbone vectors were 5373 bp, and the R10 right arm TALEN backbone vector was 4189 bp. The fragments of 4842 and 3658 bp were the sequences of TALEN backbone vectors which were simultaneously digested by the enzymes. The other fragments were the sequence of remaining TALEN backbone vectors and the concatenated sequences. The results demonstrated that the fragment lengths of the TALEN left (L1, L2, and L3) and right arm target (R1 and R2) assembly vectors were consistent with correct assembly of the vectors (Figure 2). We then confirmed the sequences that inserted into the vectors by sequencing analysis. Alignment with the reference sequence (Supplementary Figure S2) indicated that all TALEN regions (L1, L2, L3, R1, and R2) were successfully constructed and suitable for use in subsequent cellular transfection.

### Knockout of miR-192 in IPEC-J2 cells

The puromycin minimum lethal concentration for IPEC-J2 cells after 3 d was 2 μg/ml. All cells grew well for the initial 24 h after TALEN vector transfection. The control group cells started to undergo apoptosis after the media was replaced with puromycin (containing 2 μg/ml) containing media, while several cells survived in the TALEN transfected group. Any surviving cells resumed growth after refreshing the medium with complete medium without puromycin. Cellular DNA was extracted and used as template for PCR and subsequent electrophoresis results demonstrated that the product lengths were consistent with the targeting fragments (Supplementary Figure S3).

Direct sequencing found that the amplified fragment sequence was identical with the porcine genome throughout. The sequencing results for the six combinations of the TALEN vectors (L1R1, L1R2, L2R1, L2R2, L3R1, and L3R2) found that the overlapped peak existed in the miR-192 mature body region (Supplementary Figure S4), indicating that all six combinations would be expected to have targeting capability. The overlapped peak was small for the L2R1 and L2R2 combinations, compared with the other combinations, especially L3R1 and L3R2.

### Selection of miR-192 knockout monoclonal cells

We observed no significant morphological differences between the various monoclonal cells and all of the cells exhibited good proliferation. DNA was extracted from each monoclonal cell and used as template for PCR; the PCR products are shown in Supplementary Figure S5. All samples produced PCR reaction products, and the length of partial product fragments was less than 287 bp, indicating possible base deficiency among the amplified fragments, suggesting that the selected monoclonal cells would correctly target miR-192. Direct sequencing of the PCR products confirmed the presence of base deficiency and the overlapped peak within the miR-192 mature body region (Supplementary Figure S6). The efficiency of the constructs was 25% (there were 19 monoclonal cells with the base deficiency of miR-192 in total of 76 monoclonal cells).

**Figure 3:**
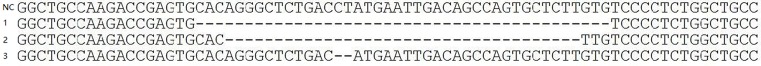
Sequence deletions in pre-miR-192 NC represents wild-type pre-miR-192 sequence; 1, 2, and 3 represent pre-miR-192 with three deletion types separately.

Those samples confirmed as having base deficiency and the overlapped peak were connected with T vectors and the monoclone selected and sent for sequencing. Three double knockout intestinal epithelial cells were identified (Figure 3). In addition, the miR-192 knockout did not affect the base composition of miR-215 (Supplementary Figure S7), indicating these TALEN design were specific for miR-192.

**Figure 4:**
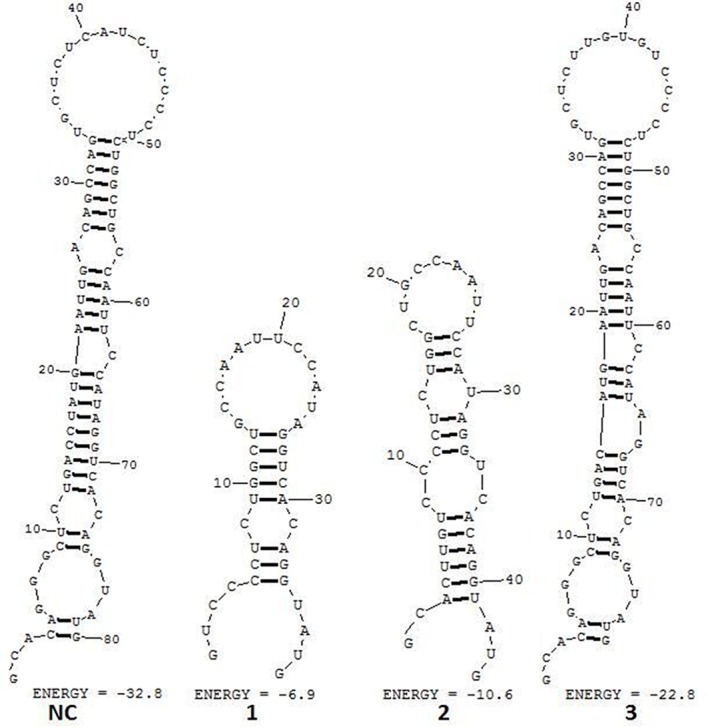
The secondary conformation of pre-miR-192 NC represents wild-type pre-miR-192 sequence; 1, 2, and 3 represent pre-miR-192 of three deletion types separately. The results of secondary conformational analysis were performed by using the RNA structure software (version 5.6). “Energy” refers to minimum free energy, and the units for the energy are kcal/mol.

Analysis of the predicted secondary structures (Figure 4) found that the precursor sequences with multiple base deficiencies would be unable to form the same secondary structure as the normal precursors, such as sequences 1 and 2. Sequence number 3 was predicted as having a 2-bp deficiency and able to form a secondary structure similar to the wild-type precursors; however, the localization of the deficient base to the mature body region leads to a failure to form the correct mature body.

### Effects analysis of miR-192 knockout on the host gene expression

**Figure 5:**
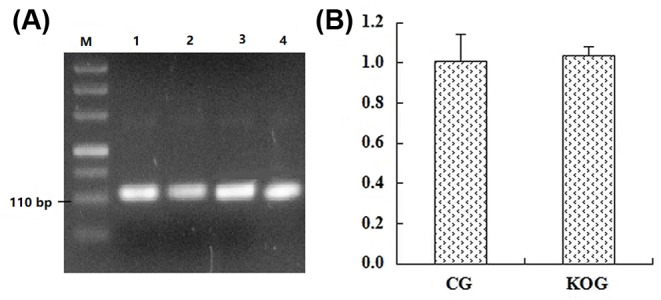
The amplification and transcription analysis of the miR-192 host’s gene (**A**) is the partial sequence amplification fragment of miR-192 host’s gene, and DL5000 DNA Marker (TaKaRa) was used for agarose gel electrophoresis. Amplification products (lanes 1 and lane 2) were from the control group (CG), and amplification products (lanes 3–4) were from knockout group (KOG). (**B**) is the transcription level of the miR-192 host’s gene from monoclonal cells.

As shown in Figure 5, the reverse transcribed product of the extracted RNA from positive knockout monoclonal cells and the control monoclonal cells were used as qRT-PCR template. The lengths of the amplification product were consistent with the targeting fragment (110 bp). Sequencing results confirmed that the base compositions of the fragments were identical with that of the database sequence (Supplementary Figure S8), indicating that the miR-192 gene knockout did not affect host gene transcription. Furthermore, our qRT-PCR data demonstrated that miR-192 gene knockout did not significantly affect host gene transcription.

### Prediction and function of miR-192 and miR-215 target genes in swine

The TargetScan predicted that the target genes of miR-192 and miR-215 are identical, miR-192 and miR-215 both has 156 potential target genes (Supplementary Table S1). Through intersection between target genes and regulatory genes (Gene Expression Omnibus, Accession number: GSE26854) for the resistance to *E. coli* F18 infection, five important target genes such as *ALCAM, DLG5, FRMD4B, MIPOL1*, and *ZFHX3* were screened out (Supplementary Table S2).

### Expression detection of five target genes predicted

**Figure 6:**
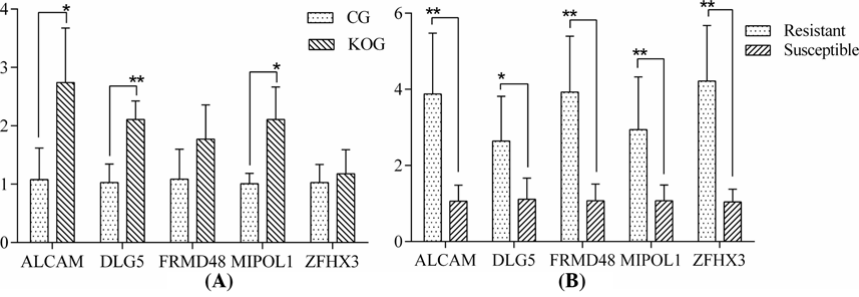
Real-time fluorescence quantitative detection results of five target genes (**A**) is the comparison between miR-192 knockout cells and control group. (**B**) is the comparison between the *E. coli* F18 disease-resistant and the *E. coli* F18-susceptible pigs. * means significant difference between two groups (*P*<0.05). ** means extremely significant difference between two groups (*P*<0.01).

**Figure 7:**
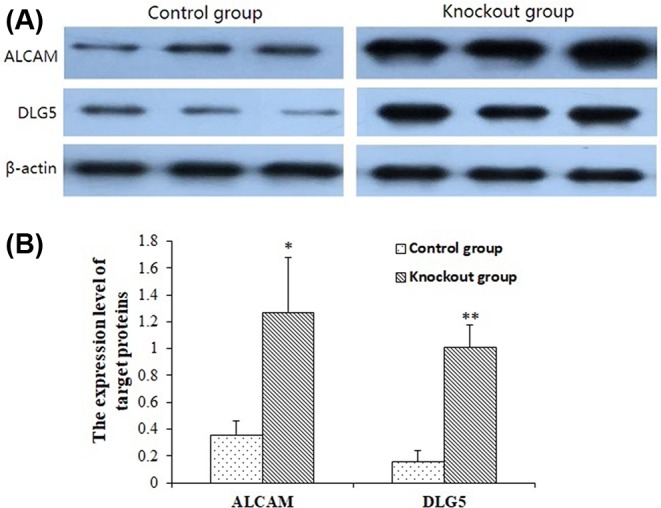
Differential expression levels of DLG5 and ALCAM proteins between miR-192 knockout cells and control group Each image shows three reduplicate samples with a similar procedure. (**A**) is the result of Western blot analysis, and (**B**) is the semi-quantitative analysis results of band gray scale, which is corrected by own gray value. The gray ratio between the band gray of the target protein and reference protein β-actin is represented as the protein expression level. * means significant difference between two groups (*P*<0.05). ** means extremely significant difference between two groups (*P*<0.01).

QPCR detection results of five target genes are shown in Figure 6. As shown in Figure 6A, the expression levels of five target genes in knockout cells were higher than control group cells. Thereinto, the expression level of *DLG5* was extremely significantly different between miR-192 knockout cells and control group, and the expression levels of *ALCAM* and *MIPOL1* were significantly different between miR-192 knockout cells and control group. In Figure 6B, the relative expression of five target genes in resistant individuals was higher than that of sensitive individuals, and reached significant level (*P*<0.05). Further Western blotting analysis showed that the translational levels of DLG5 and ALCAM in miR-192 knockout cells were higher than the control group (Figure 7).

### Effects of miR-192 knockout on *E. coli* adhesion ability for IPEC-J2 cell

**Figure 8:**
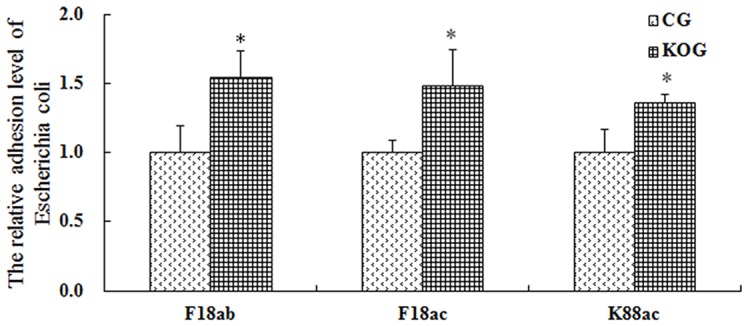
Bacterial adhesion levels in the miR-192 knockout cells CG represents the control group and KOG represents the miR-192 knockout group. * means significant difference between two groups (*P*<0.05). ** means extremely significant difference between two groups (*P*<0.01).

Bacterial adhesion assay results showed that the adhesion ability of the *E. coli* F18ab, F18ac, and K88ac cells improved after miR-192 knockout, and the adhesion ability of the *E. coli* F18ab, F18ac, and K88ac cells increased to more than 1-fold change improved after miR-192 knockout (Figure 8).

## Discussion

The low efficiency of traditional targeting technology has resulted in its limited application. Zinc finger nucleases (ZFN) technology has drawbacks, including complex experiment handling, high cost, and low specificity [19]. Whereas TALEN technology has simple experiment handling, is relatively rapid and has high sequence specificity, high shear efficiency (10–50%), and low off-target efficiency [20]. The only limiting factor of TALEN is that the first base prior to the recognition site must be thymine. Compared with the preceding two methods, CRISPR (Clusteredregularly interspaced short palindromic repeat) has simpler and faster construction, lower cost and higher editing efficiency, and can realize multiple gene targeting at the same time [21]. But the off-target efficiency is higher, and there is nonspecific cleavage phenomenon. The TALEN method was used to edit the gene in the present study. TALEN has been successfully applied to zebrafish, mice, pigs, and sheep [22–24]. In addition, TALEN technology has been used for the fixed point knockout of noncoding RNA (e.g. miRNA) in human and mouse studies [25,26]. However, few reports have applied TALEN technology to miRNA knockout in pigs [27,28]. Here, we successfully constructed a TALEN recognition vector consisting of three left arms and two right arms and transfected a total of six left/right arm combinations into porcine intestinal epithelial cells (IPEC-J2). We found that specific targeting to the miR-192 mature body sequences could be achieved with all six combinations but that differences existed in the efficiency of the arm combinations.

Fixed point knocking out of miRNA requires a different approach to that used for knocking out of a protein encoding gene. For protein encoding gene knockout, a base deletion combined with frame shift mutations or early emergence of a termination codon are sufficient for successful gene knockout. Base deletion or functional loss of the mature body is necessary for miRNA knockout [26,29]. The TALEN vector designed in the present study was able to specifically target miR-192, resulting in deletion or insertion errors during the DNA repair process. Thus, three miR-192 double tapping cells were obtained by screening and culturing of monoclonal cells. Despite miR-192 having a role in cell cycle regulation, the miR-192 double-tapped intestinal epithelial cells were able to grow normally, suggesting that miR-192 is not necessary for the growth of intestinal epithelial cells [30]. There are more bases missing from the miR-192 mature regions of the double-tapped cells, which lead to a failure in proper stem formation when the miR-192 precursor forms a hairpin structure. The mature miRNA sequence is positioned at the stem of the miRNA precursor hairpin structure, thus, even if the hairpin structure is formed, the correct sequence of the mature miRNA cannot be excised. A large part of the mammalian miRNA genes are derived from the introns of protein encoding genes [31,32], as is the case for miR-192. Despite the detection of miR-192 transcripts in the wild-type host cells, knockout of the miR-192 had no effect on the expression of nearby genes, thus providing a reliable cell model in which to explore miR-192 function.

Regarding the molecular mechanism of F18 *E. coli* resistance in swine, Vogeli et al. [33] found that G/A mutation at the M307 locus of the α(1,2) fucosyltransferase (*FUT1*) encoding gene can be used as a genetic marker for ETEC F18 resistance in European pigs. The pig with genotype AA is resistant to *E. coli* 18, while the pig with genotype GG and AG are susceptible. Meijerink et al. [34] subsequently carried out resistance breeding in the Switzerland Yorkshire, Duroc, Hampshire and Pietrain breeds based on this marker. However, only the GG genotype was detected at the *FUT1* gene M307 locus of wild boar breeds and more than 20 Chinese native pig breeds, which extremely skewed the distribution [35]. Western pig breeds originated from European boars, while Chinese native pig breeds originated from Asian wild boars. In comparison with Western commercial pig breeds, Chinese native pig breeds are generally considered as owning better performances on resisting post-weaning diarrhea in piglets. Moreover, different Chinese native pig breeds have different resistant performance. However, nearly all of Chinese native pig breeds only presented the susceptible genotype GG at M307 in *FUT1*, and were absent of the genetic background of *FUT1* on the resistance to *E. coli* F18. The *FUT1* gene markers are therefore unsuitable for Chinese native pig breeds. The genetic basis of resistance to *E. coli* F18 infection might be different for Chinese and Western pig breeds; therefore, it is important to screen for important candidate genes and effective genetic markers for *E. coli* F18 resistance in Chinese pig breeds. Our data suggest that miR-192 has a role in *E. coli* resistance in weaned pigs, which is in line with previous high-throughput sequencing and quantitative tests. Using TELEN technology, we found that the *E. coli* adhesion capability improved after miR-192 knockout, further supporting the importance of miR-192 in *E. coli* resistance. Nevertheless, the precise role of miR-192 remains unclear. We predicted the gene targets of miR-192 and preliminary screened five likely candidates (*DLG5* (Discs large homolog 5), *ALCAM* (Activated leukocyte cell adhesion molecule), *FRMD4B* (FERM domain containing 4B), *MIPOL1* (Mirror-image polydactyly 1), and *ZFHX3* (Zinc finger homeobox 3)) related to *E. coli* F18. We found the expression levels of target genes in knockout cells were higher than control group, the expression levels of *DLG5, ALCAM*, and *MIPOL1* were significantly different between miR-192 knockout cells and control group. At the same time, the expression of five target genes in resistant individuals was significantly higher than that of sensitive individuals. DLG5 is a membrane associated guanylate kinase protein family member and localized in regions of cell connections. DLG5 can form a variety of complexes with other proteins and plays an important role in regulating cell growth, cell migration, maintaining the structural integrity of the epithelial cells, and signal transmission; DLG5 is associated with inflammatory bowel disease [36]. ALCAM belongs to the immunoglobulin superfamily, involved in tumorigenesis, inflammation, hematopoietic stem cell differentiation, and cell adhesion [37]. FRMD4B may be a skeleton protein, and participates in the establishment of epithelial cell polarity [38]. MIPOL1 is mainly associated with body hypotrophy and tumor inhibitory [39,40]. ZFHX3 is a transcription factor of zinc ring structure, can be combined with AT-rich enhancer motif, also is called ATBF1 (AT-motif binding factor 1), involved in embryonic development, neuronal differentiation, response to DNA damage and thermal stress, and tumor inhibitory [41–44], and interacts with PIAS3 to regulate STAT3-mediated signaling transduction pathways [45]. Based on the functional analysis of five important target genes, *DLG5* and *ALCAM* play an important role in cell adhesion, inflammatory response, cell polarity and cell morphology, especially DLG5’s role in maintaining the structural integrity of intestinal epithelial cells, so we can deduce that *DLG5* and *ALCAM* could be key target genes for miR-192 and thus account for miR-192’s role in *E. coli* infection, and the high expression of *DLG5* and *ALCAM* conduces to maintain the stability of the cell structure and reduce the infection of *E. coli.* MiR-192 and its key target genes are also important for maintaining normal intestinal function in weaned piglets.

Based on our findings, we propose that the miR-192 deficiency intestinal epithelial cells generated here should be used in further proteomic studies, which would likely improve our understanding of the regulatory mechanisms of *E. coli* resistance in pigs.

## Conclusion

The present study established successfully porcine intestinal epithelial cells defective in miR-192, and illustrated that miR-192 knockout can enhance the adhesion ability of the *E. coli* strains F18ab, F18ac, and K88ac, meanwhile increase the expression of target genes (*DLG5* and *ALCAM*). The results suggested that miR-192 and its key target genes (*DLG5* and *ALCAM*) could have a key role in *E. coli* infection in piglets.

## Supporting information

**Figure S1:** The integral workflow of producing the intestinal epithelial cells with miR-192 knockout through TALEN technology.

**Figure S2:** The comparison results of the sequencing and the standard sequence for TALEN vectors of left and right arms. According to the comparison results, it was saw that TALEN vectors were successfully constructed.

**Figure S3:** PCR products of miR-192 detected by agarose gel electrophoresis. These represents the reaction products when using template DNA from untreated (NC) IPEC-J2 cells and cells transfected and screened using the L1R1, L1R2, L2R1, L2R2, L3R1 and L3R2 TALEN vectors. M represents 100bp Ladder II (Beijing Dingguo Changsheng Biotechnol Co.Ltd.).

**Figure S4:** Sequencing analysis of the PCR amplified products of porcine miR-192 region with different treatments different. The sequencing results for the six combinations of TALEN vectors (L1R1, L1R2, L2R1, L2R2, L3R1 and L3R2) found that the overlapped peak existed in the miR-192 mature body region.

**Figure S5:** PCR products detection of miR-192 region in monoclonal cells. M represents DL500 DNA Marker (Takara Biomedical Technology (Beijing) Co., Ltd. The monoclonal cells (lane 4, 7, 12, 13) may be defective in miR-192.

**Figure S6:** Sequencing analysis of the miR-192 PCR amplified products from monoclonal cell. © was untreated cells, and 1, 2, 3 were defective in miR-192.

**Figure S7:** Sequencing analysis of the miR-192 PCR amplified products from monoclonal cell. CG was control group, and KG represents the miR-192 knockout group.

**Figure S8:** Sequencing analysis of qRT-PCR amplified products of miR-192 host gene from monoclonal cell. CG was control group, and KG represents the miR-192 knockout group.

**Table S1:** Related information of miR-192 and miR-215 target genes.

**Table S1:** Specific information of important target genes.

## Abbreviations

*ALCAM*: activated leukocyte cell adhesion molecule
*DLG5*: Discs large homolog 5
*E. coli*: *Escherichia coli*
ETEC: enterotoxigenic *Escherichia coli*
*FRMD4B*: FERM domain containing 4B
*FUT1*: α(1,2) fucosyltransferase
IPEC-J2: porcine intestinal epithelial cells
*MIPOL1*: Mirror-image polydactyly 1
miRNA: microRNA
qRT-PCR: quantitative real-time PCR
TALEN: transcription activator-like effector nuclease
UTR: untranslated regions
*ZFHX3*: zinc finger homeobox 3
ZFN: zinc finger nucleases
