# Supplementary material for: Insight into the molecular mechanism of miR-192 regulating Escherichia coli resistance in piglets
Source: Biosci Rep. 2018 Feb 21;38(1):BSR20171160. doi: 10.1042/BSR20171160 (PMC5821941; doi:10.1042/BSR20171160)
Supplement: Supplementary file 1 [file bsr20171160_Supp1.pdf]

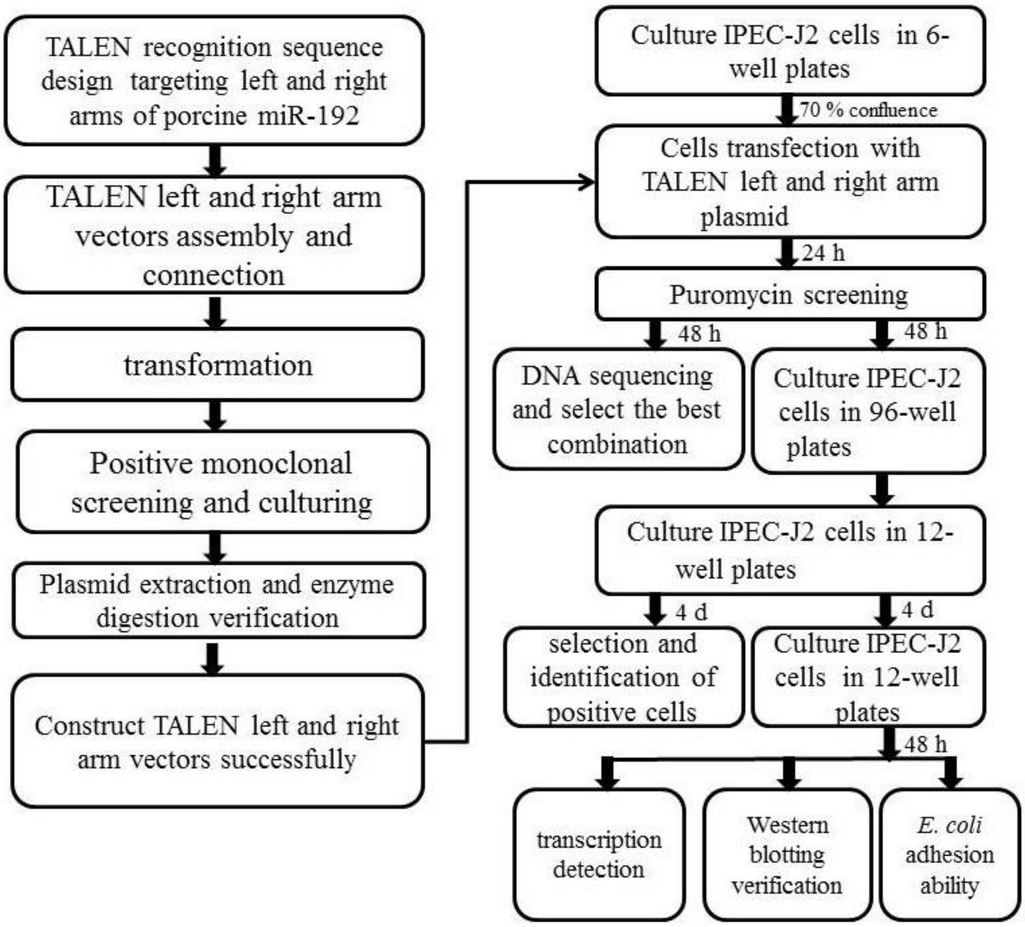

Figure S1. The integral workflow of producing the intestinal epithelial cells with miR-192 knockout through TALEN technology.

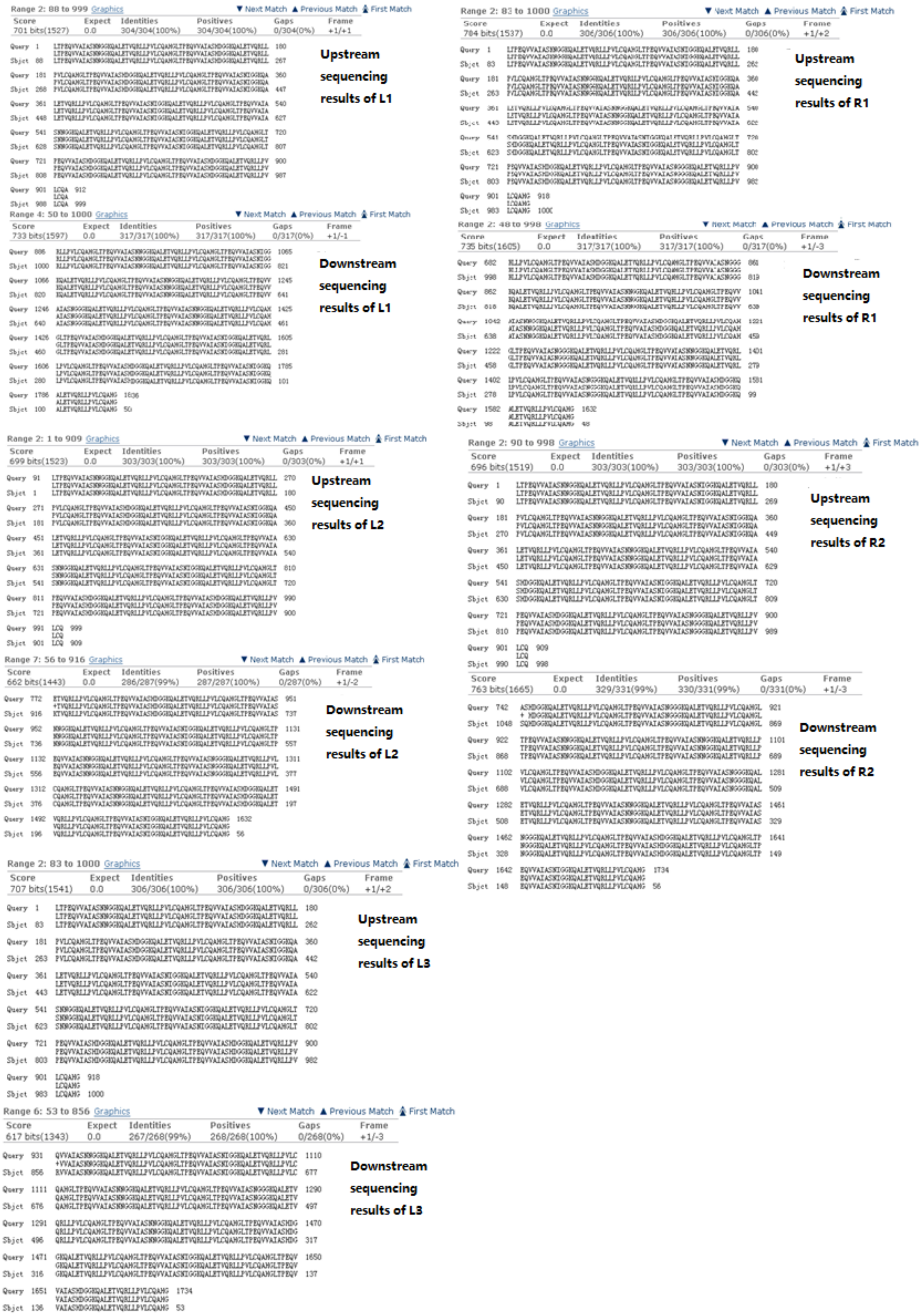

Figure S2. The comparison results of the sequencing and the standard sequence for TALEN vectors of left and right arms. According to the comparison results, it was saw that TALEN vectors were successfully constructed.

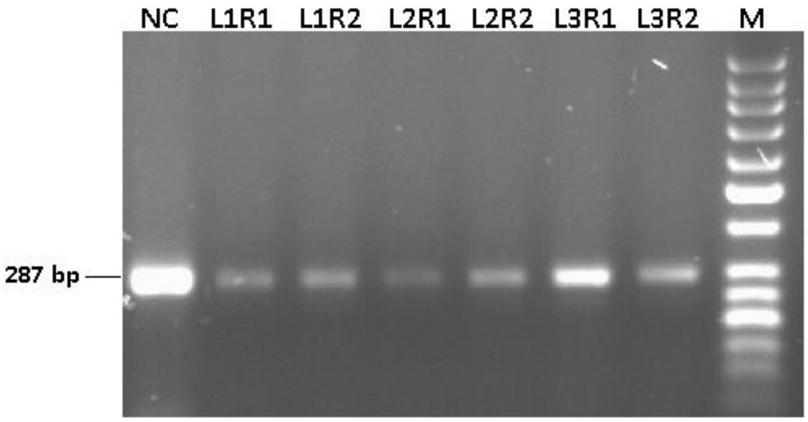

Figure S3. PCR products of miR-192 detected by agarose gel electrophoresis. These represents the reaction products when using template DNA from untreated (NC) IPEC-J2 cells and cells transfected and screened using the L1R1, L1R2, L2R1, L2R2, L3R1 and L3R2 TALEN vectors. M represents 100bp Ladder Ⅱ(Beijing Dingguo Changsheng Biotechnol Co.Ltd.).

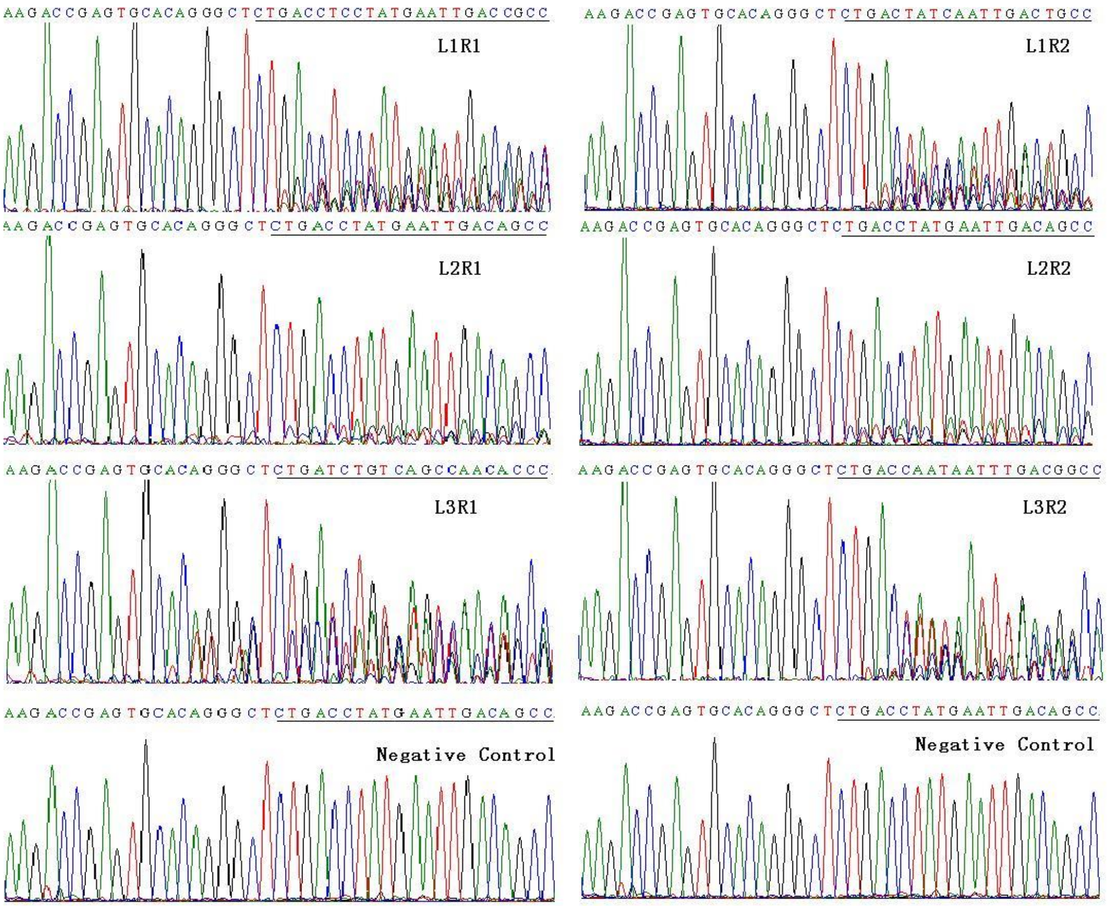

Figure S4. Sequencing analysis of the PCR amplified products of the porcine miR-192 region with different treatments. The sequencing results for the six combinations of the TALEN vectors (L1R1, L1R2, L2R1, L2R2, L3R1 and L3R2) found that the overlapped peak existed in the miR-192 mature body region.

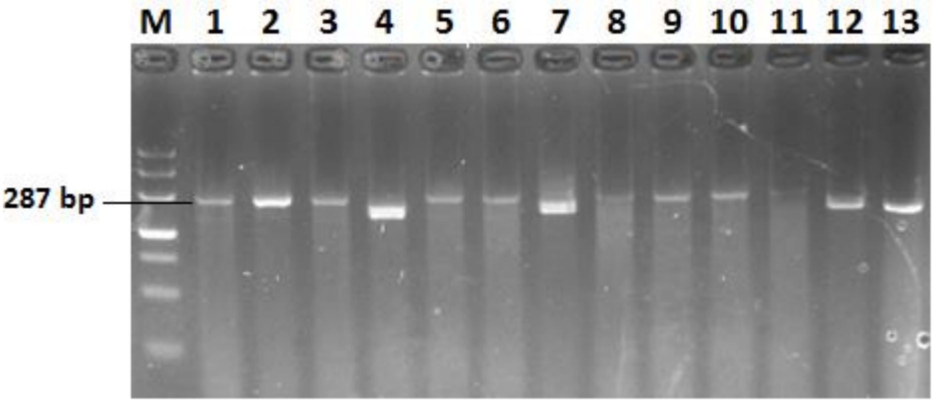

Figure S5. PCR products detection of miR-192 region in monoclonal cells. M represents DL500 DNA Marker (Takara Biomedical Technology (Beijing) Co., Ltd.). The monoclonal cells (lane 4, 7, 12, 13) may be defective in miR-192.

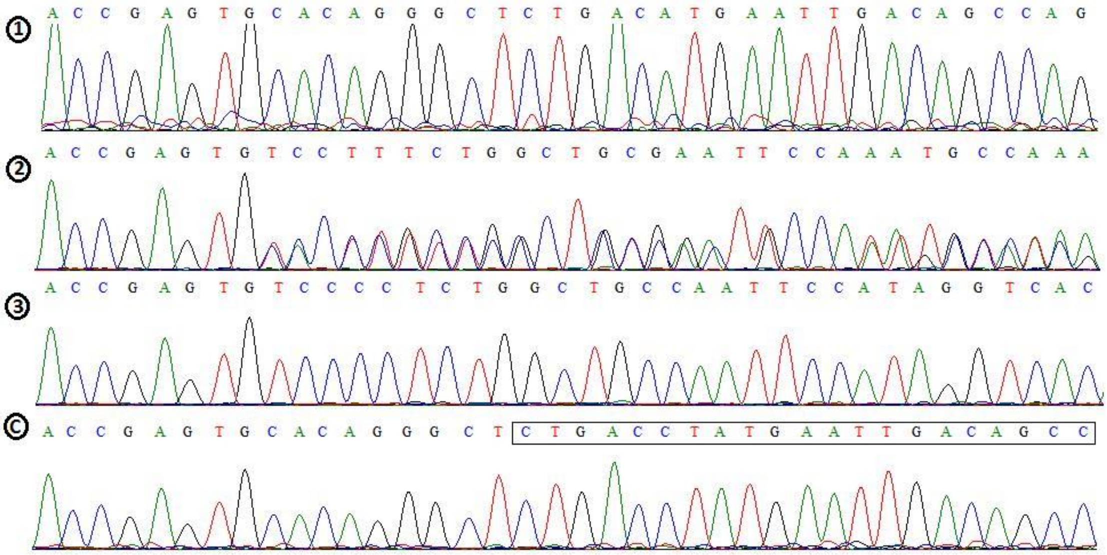

Figure S6. Sequencing analysis of the miR-192 PCR amplified products from monoclonal cell.© was untreated cells, and ①, ②, ③ were defective in miR-192.

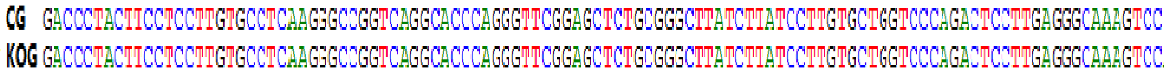

Figure S7. Sequencing analysis of the miR-215 PCR amplified products from monoclonal cell. CG was control group, and KG represents the miR-192 knockout group.

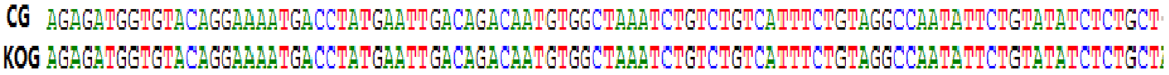

Figure S8. Sequencing analysis of qRT-PCR amplified products of miR-192 host gene from monoclonal cell. CG was control group, and KG represents the miR-192 knockout group.

Table S1 Related information of miR-192 and miR-215 target genes.

Gene name	transcript	Gene name	transcript	Gene name	transcript
PABPC4	NM_001135653	*SRSF6*	NM_006275	*ASXL2*	NM_018263
CCNT2	NM_001241	*KIDINS220*	NM_020738	*SHISA9*	NM_001145204
ARFGEF1	NM_006421	*WSCD2*	NM_014653	*PCDH19*	NM_001105243
EREG	NM_001432	*ZFHX3*	NM_001164766	*ZBTB34*	NM_001099270
LPAR4	NM_005296	*ATF1*	NM_005171	*RUNX1T1*	NM_001198625
BHLHE22	NM_152414	*PDP1*	NM_001161778	*FAM19A3*	NM_001004440
PRKAR1A	NM_002734	*PTPRT*	NM_007050	*KLHL15*	NM_030624
KIF20B	NM_016195	*HIGD1A*	NM_001099668	*SIK1*	NM_173354
RPAP2	NM_024813	*ENC1*	NM_003633	*TMTC3*	NM_181783
SLITRK4	NM_001184749	*ZNF652*	NM_001145365	*ENY2*	NM_001193557
IKZF2	NM_001079526	*KPNA4*	NM_002268	*GLP1R*	NM_002062
PLXNB2	NM_012401	*RGMB*	NM_001012761	*CCDC152*	NM_001134848
ACPP	NM_001099	*C15orf24*	NM_020154	*ARHGAP36*	NM_144967
TYMS	NM_001071	*CTCF*	NM_001191022	*UBE2QL1*	NM_001145161
RAB2A	NM_001242644	*C9orf100*	NM_032818	*FNDC3B*	NM_001135095
FRMD4B	NM_015123	*WNK1*	NM_001184985	*RUNX1*	NM_001001890
C6orf225	NM_001033564	*IER5*	NM_016545	*IGDCC4*	NM_020962
DBT	NM_001918	*TRPM7*	NM_017672	*ACVR2A*	NM_001616
DICER1	NM_001195573	*ANKRD44*	NM_001195144	*TIFA*	NM_052864
MIPOL1	NM_001195296	*C4orf3*	NM_001001701	*FAM167A*	NM_053279
PKP4	NM_001005476	*CXCR5*	NM_001716	*PIK3IP1*	NM_001135911
DNAH5	NM_001369	*TCEB3*	NM_003198	*NCOA3*	NM_001174087
MFAP3	NM_001135037	*C8orf46*	NM_152765	*TSHZ2*	NM_001193421
KIAA0754	NM_015038	*SH3RF3*	NM_001099289	*EIF5A2*	NM_020390
ZEB2	NM_001171653	*NIPBL*	NM_015384	*ARHGAP19*	NM_001204300
PRKD3	NM_005813	*PPP1R3D*	NM_006242	*COL5A1*	NM_000093
C6orf168	NM_032511	*GMEB1*	NM_006582	*DOK6*	NM_152721
ZNF536	NM_014717	*ACTBL2*	NM_001017992	*IGF1*	NM_000618

CHD7	NM_017780	*CNPY1*	NM_001103176	*ARFIP2*	NM_001242854
MSN	NM_002444	*GRHL1*	NM_198182	*LMTK2*	NM_014916
SYT6	NM_205848	*SH2B3*	NM_005475	*VPS53*	NM_001128159
AP3M2	NM_001134296	*RICTOR*	NM_152756	*LYRM7*	NM_181705
WDR44	NM_001184965	*PARP8*	NM_001178055	*DCC*	NM_005215
C20orf108	NM_080821	*TRERF1*	NM_033502	*CBL*	NM_005188
ALCAM	NM_001627	*GDF11*	NM_005811	*TAOK1*	NM_020791
TDG	NM_003211	*PDSS2*	NM_020381	*RAP1GAP2*	NM_001100398
CLSTN1	NM_001009566	*IGDCC3*	NM_004884	*RSAD2*	NM_080657
NSF	NM_006178	*HOOK3*	NM_032410	*CUX1*	NM_001202543
LRRFIP1	NM_001137552	*ZPBP2*	NM_198844	*SRGAP3*	NM_001033117
FAM123B	NM_152424	*NKAIN2*	NM_001040214	*NFAT5*	NM_001113178
CREB5	NM_001011666	*SMC5*	NM_015110	*ABHD2*	NM_007011
ACVR2B	NM_001106	*PITPNB*	NM_012399	*PAIP2B*	NM_020459
H3F3B	NM_005324	*FOXN1*	NM_003593	*SIRT5*	NM_031244
OLIG3	NM_175747	*XPO4*	NM_022459	*SLC5A3*	NM_006933
ANAPC16	NM_001242546	*FABP3*	NM_004102	*CPEB4*	NM_030627
BLCAP	NM_001167820	*GDAP2*	NM_017686	*SHANK2*	NM_012309
CTNNBIP1	NM_001012329	*C4orf46*	NM_001008393	*YAF2*	NM_001190980
RPRD1B	NM_021215	*NAA50*	NM_025146	*CDON*	NM_016952
DLG5	NM_004747	*TCTEX1D1*	NM_152665	*PGM3*	NM_001199917
SLC39A6	NM_012319	*PHTF2*	NM_001127357	*WWC2*	NM_024949
BRD3	NM_007371	*KPNA6*	NM_012316	*UCHL5*	NM_001199261
KLHDC5	NM_020782	*ARL4C*	NM_005737	*ATF7*	NM_001130059

Table S2 Specific information of important target genes.

Target gene	Representative transcript	Gene name
DLG5	NM_004747	Discs, large homolog 5
ALCAM	NM_001627	Activated leukocyte cell adhesion molecule
ZFHX3	NM_001164766	Zinc finger homeobox 3
FRMD4B	NM_015123	FERM domain containing 4B
MIPOL1	NM_001195296	Mirror-image polydactyly 1
